# High Gamma and Beta Temporal Interference Stimulation in the Human Motor Cortex Improves Motor Functions

**DOI:** 10.3389/fnins.2021.800436

**Published:** 2022-01-03

**Authors:** Ru Ma, Xinzhao Xia, Wei Zhang, Zhuo Lu, Qianying Wu, Jiangtian Cui, Hongwen Song, Chuan Fan, Xueli Chen, Rujing Zha, Junjie Wei, Gong-Jun Ji, Xiaoxiao Wang, Bensheng Qiu, Xiaochu Zhang

**Affiliations:** ^1^Hefei National Laboratory for Physical Sciences at the Microscale, Division of Life Science and Medicine, Department of Radiology, The First Affiliated Hospital of USTC, School of Life Science, University of Science and Technology of China, Hefei, China; ^2^Centers for Biomedical Engineering, School of Information Science and Technology, University of Science and Technology of China, Hefei, China; ^3^Division of the Humanities and Social Sciences, California Institute of Technology, Pasadena, CA, United States; ^4^School of Optometry and Vision Sciences, Cardiff University, Cardiff, United Kingdom; ^5^Department of Neurology, The First Affiliated Hospital of Anhui Medical University, Hefei, China; ^6^Department of Psychology, School of Humanities and Social Science, University of Science and Technology of China, Hefei, China; ^7^Biomedical Sciences and Health Laboratory of Anhui Province, University of Science and Technology of China, Hefei, China

**Keywords:** temporal interference stimulation, non-invasive brain stimulation, brain oscillation, motor function, motor cortex excitability

## Abstract

**Background:** Temporal interference (TI) stimulation is a new technique of non-invasive brain stimulation. Envelope-modulated waveforms with two high-frequency carriers can activate neurons in target brain regions without stimulating the overlying cortex, which has been validated in mouse brains. However, whether TI stimulation can work on the human brain has not been elucidated.

**Objective:** To assess the effectiveness of the envelope-modulated waveform of TI stimulation on the human primary motor cortex (M1).

**Methods:** Participants attended three sessions of 30-min TI stimulation during a random reaction time task (RRTT) or a serial reaction time task (SRTT). Motor cortex excitability was measured before and after TI stimulation.

**Results:** In the RRTT experiment, only 70 Hz TI stimulation had a promoting effect on the reaction time (RT) performance and excitability of the motor cortex compared to sham stimulation. Meanwhile, compared with the sham condition, only 20 Hz TI stimulation significantly facilitated motor learning in the SRTT experiment, which was significantly positively correlated with the increase in motor evoked potential.

**Conclusion:** These results indicate that the envelope-modulated waveform of TI stimulation has a significant promoting effect on human motor functions, experimentally suggesting the effectiveness of TI stimulation in humans for the first time and paving the way for further explorations.

## Introduction

Electrical stimulation is the most direct way to regulate neuroplasticity and electrically oscillating neural activities (Doty, 1969). Two kinds of electrical stimulation techniques have been extensively used. The first is deep brain stimulation (DBS), which has been proven to be an effective treatment for treating Parkinson’s disease (Benabid, 2003; Tinkhauser et al., 2017). The delivery of DBS requires invasive surgery, thus presenting the potential for surgical complications (Ramirez-Zamora et al., 2018). Another method is transcranial electrical stimulation (tES) (Paulus, 2011; Bestmann and Walsh, 2017), which can modulate brain activities in non-invasive ways (Keeser et al., 2011; Yang et al., 2014, 2017, 2020; Liu et al., 2018). Transcranial electrical stimulation applied with alternating current, i.e., transcranial alternating current stimulation (tACS), has been used to facilitate oscillation activity within specific frequency ranges (Herrmann et al., 2013; Vossen et al., 2015). Many studies have shown that tACS can modulate motor-related oscillation brain activities, which could result in changes in cortical excitability and motor function improvement (Feurra et al., 2011, 2013; Joundi et al., 2012; Pollok et al., 2015). However, currents of tES applied over the scalp were found to be significantly attenuated when traveling through the skin, subcutaneous soft tissue and skull (Vöröslakos et al., 2018). Thus, the depth of stimulation is limited. Although simulation and experimental results show that conventional tES could also generate an electric field with enough strength to influence neural activity in some regions deep in the brain (Huang and Parra, 2019; Louviot et al., 2021). But the intensity of the electric field in the brain regions that cover the deep target would be larger (Lee et al., 2020), which might cause off-target effects and make the results difficult to explain.

To overcome the limitations of these two electrical brain stimulation techniques, temporal interference (TI) stimulation has been recently proposed (Grossman et al., 2017), which has caused considerable excitement in the research community (Dmochowski et al., 2017; Lozano, 2017; Opitz and Tyler, 2017; Grossman, 2018; Grossman et al., 2018; Halpern et al., 2018). This new technique can be applied by delivering two electric fields at frequencies that are too high (≥1 kHz) to entrain neural electrical activity (Hutcheon and Yarom, 2000). The frequency difference between these two electric fields is within the range of brain oscillations (e.g., 20 Hz, 70 Hz, etc.), which could result in a prominent envelope modulated electric field in a targeted brain region. TI stimulation has been proven to be effective in driving the firing patterns of hippocampal neurons without recruiting neurons in the overlying brain cortex and evoking different motor behaviors when targeting different areas of the motor cortex in mice (Grossman et al., 2017).

Based on the concept of TI stimulation, several modeling and computation studies have been performed to explore the feasibility of TI stimulation in the human brain (Lafon et al., 2017; Fariba et al., 2019; Rampersad et al., 2019; Cao and Grover, 2020; Huang et al., 2020; Lee et al., 2020; Mirzakhalili et al., 2020). However, no data about the actual effect of TI stimulation on human brains have been reported thus far. The stimulation waveform of TI stimulation is an envelope-modulated waveform produced by the superposition of two sine waves, which is much more complex than conventional tACS. Whether such TI stimulation has a comparable effect with conventional tACS on the human brain is unknown.

In this study, we implemented TI stimulation targeting the left primary motor cortex (M1) of healthy participants to validate the effectiveness of TI stimulation on the human brain. The primary motor cortex (M1) is a common target in many pioneering experiments in non-invasive brain stimulation (NIBS) technique (Barker et al., 1985; Priori et al., 1998; Antal et al., 2008; Terney et al., 2008). High gamma and beta brain oscillations play important roles in human motor cortex. Previous studies have found that high gamma brain oscillations (e.g., 70 Hz) are transiently increased during movement and they have a promoting effect on movement initiation (Cheyne et al., 2008; Muthukumaraswamy, 2010; Gaetz et al., 2013). Meanwhile, beta activities (e.g., 20 Hz) in the motor cortex are considered an important component of motor learning (Espenhahn et al., 2019, 2020; Haar and Faisal, 2020).

Considering the prior investigations of oscillations related to M1, we designed two stimulation conditions with envelope frequencies of 20 Hz (beta) and 70 Hz (high gamma). A sham condition was used as a control. To explore the influence of TI stimulation on different levels of motor functions, two motor tasks were employed in two independent experiments, including a random reaction time task (RRTT) and a serial reaction time task (SRTT). RRTT is a single reaction time task, and the order of the reactions is totally randomized. SRTT contains repeatedly recurring response sequences, which can be learned by participants (Robertson, 2007). Based on the distinct functions of high gamma and beta oscillations in the human motor cortex we stated above, we hypothesized a promotion of reaction speed induced by 70 Hz TI stimulation in RRTT and a more significant effect of 20 Hz TI stimulation than sham stimulation on motor learning in SRTT. We also measured motor cortex excitability before and after TI stimulation (Chen, 2000; Rossini et al., 2015), which was hypothesized to be facilitated by TI stimulation based on previous findings (Moliadze et al., 2010; Feurra et al., 2011, 2013; Guerra et al., 2020).

## Materials and Methods

### Participants

We recruited 27 healthy adult volunteers in the RRTT experiment, and 6 participants were removed from the analysis because of technical issues (a decrease in current due to poor contact and current crosstalk due to the flow of conductive paste). Data from the remaining 21 participants were included in the analysis (6 females, mean age ± SD: 22.429 ± 2.249 years, mean education level ± SD: 15.762 ± 2.166 years, mean handedness score ± SD: 86.667 ± 17.127). Another 33 healthy adults volunteered to participate in the SRTT experiment, but 1 participant was removed due to the sliding of electrodes, 1 participant was rejected because he switched his performing hand, and 2 participants’ data were removed because of technical issues (current crosstalk due to the flow of conductive paste). Therefore, data of 29 participants remained to be analyzed in the SRTT experiment (15 females, mean age ± SD: 22.103 ± 2.024 years, mean education level ± SD:15.966 ± 1.991 years, mean handedness score ± SD: 77.672 ± 23.792).

All participants reported no history of craniotomy or injury to the head, no personal or family history of neurological or psychiatric disease, no metal implants or implanted electronic devices, no skin sensitivity and no use of medicine during the experiment. For safety reasons, any participant who was pregnant or could be pregnant was rejected. All participants were right-handed as assessed using the Edinburgh handedness inventory (Oldfield, 1971) and had normal or corrected-to-normal vision. Informed consent was obtained prior to any involvement in the study. This study was approved by the Human Ethics Committee of the University of Science and Technology of China (IRB Number: 2020KY161).

Sample size was calculated by G*Power 3.1 (Faul et al., 2009). According to previous studies (Joundi et al., 2012; Pollok et al., 2015), we expected an effect size a little higher than medium level (Cohen’s *d* = 0.6) for the paired *t* test between stimulation conditions (20 Hz / 70 Hz) and sham condition. With α error probability of 0.05 and power (1-β error probability) of 0.8, the resulting sample size was 24. Considering potential dropouts, we recruited a bit more participants.

### Experimental Design

**FIGURE 1:**
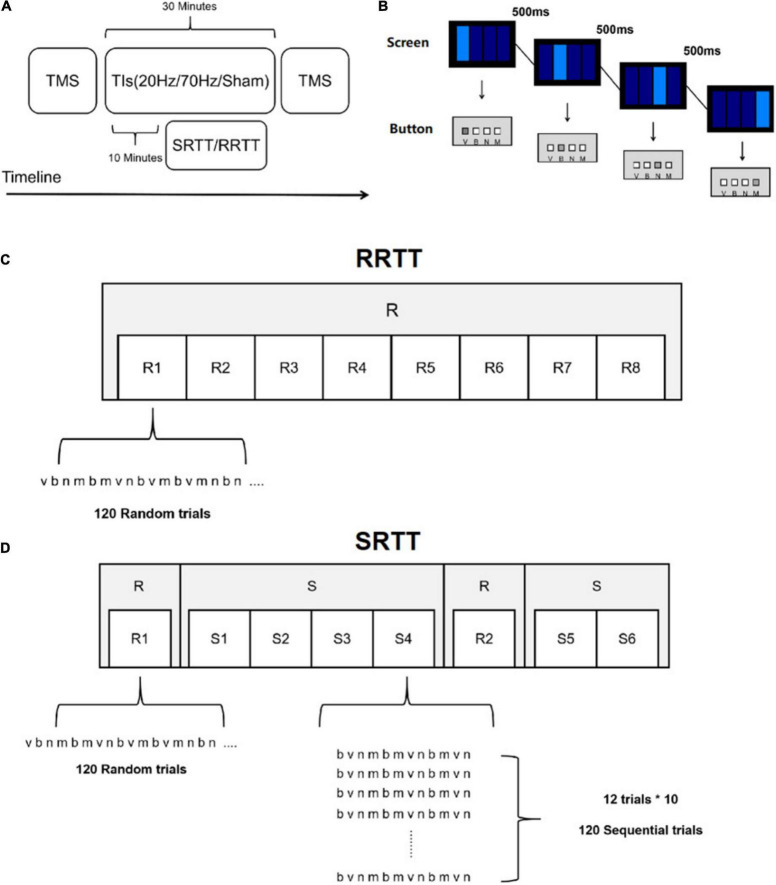
Experimental design and motor tasks. **(A)** The experimental procedures. **(B)** Motor task implemented in our experiments. **(C)** RRTT. **(D)** SRTT. In R blocks, there were three 12-item (bnmvnbmnvbvm, nvnmbvmnbmvb, mvbmnbnvmbvn) sequences with comparable difficulty for each experimental session in a counterbalanced way.

Three conditions, 20 Hz, 70 Hz and sham were applied in a single-blind, cross-over design (Figure 1A) in both the RRTT and the SRTT experiment. Participants visited the laboratory three times, at least 3 days apart, to avoid any influence of the carry-over effects of stimulation. The order of the stimulation condition (20 Hz/70 Hz/sham) was counterbalanced across participants. At the beginning of the procedures, individual M1 location was identified by single pulse TMS, and baseline motor cortex excitability was measured. Before stimulation, the participants were asked to perform a practice task with 24 random button presses. Formal experimental tasks (RRTT or SRTT) started 10 min after the beginning of TI stimulation. After the 30-min stimulation, motor cortex excitability was measured again to detect the change in excitability of M1.

### TMS and MEP

Single pulse TMS was delivered manually using a figure-eight coil (AirFile Coil) connected to a Magstim Rapid^2^ stimulator (The Magstim Company Ltd., Whitland, United Kingdom). Peak magnetic field of the coil is 0.8 T. To ensure the position of the coil to be consistent during the experiment, a Brainsight navigation system (Rogue Research Inc., Montreal, QC, Canada) was used. Electromyogram (EMG) of the right FDI was recorded by a pair of disposable self-adhesive electrodes in a belly tendon montage using the EMG module of the navigation system. The two electrodes were located over the muscle body of the right FDI and the first phalanx of the right index finger, respectively. Another electrode was attached at the underside of the right forearm as the ground electrode. EMG was recorded with a sample rate of 3 kHz and bandpass filtered at 16–470 Hz in Brainsight v2.3.8 software.

FDI hotspot was defined as the coil location steadily eliciting MEPs with the lowest stimulation intensity. The search for the FDI hotspot begun at the scalp location corresponding to C3 in the electroencephalography (EEG) 10–20 system. The initial TMS output intensity was set at 40% of the maximum stimulator output. The coil was placed tangentially over the scalp, and the handle of the coil was pointing posterolaterally 45° from the midline (Sakai et al., 1997; Opitz et al., 2013). Single-pulse TMS stimulation was manually triggered while we gradually moved the coil around the initial position. The search procedure was repeated with the stimulator output intensity increased in a 5% step until the TMS pulse could elicit any detectable MEP. In order to restrict the hotspot area, the TMS intensity was decreased by a staircase approach to diminish the current spread of the stimulation after the location that could the highest MEPs robustly be elicited was found (Raco et al., 2017). The FDI hotspot was then marked on a medical elastic bandage on the participants’ heads after the search process. The resting motor threshold (RMT) was defined as the lowest stimulus intensity that could elicit a MEP in the resting muscle with an amplitude of 50 μV (peak-to-peak) or greater in at least 5 out of 10 recordings (Boroojerdi et al., 2001; Rossini et al., 2015).

In the RRTT experiment, we applied 15 pulses over the FDI hotspot with an interval of 7 s at stimulation intensities of 120, 100, 130, 110, and 140% of RMT before and after TI stimulation (Kleim et al., 2007). We measured 30 MEPs at a stimulation intensity of 120% of the RMT in the SRTT experiment. Only 120% RMT was used because this intensity corresponds to the linear increase range of the IO curve and is sensitive to the change in M1 excitability (Rossini et al., 2015).

### Motor Tasks

The motor tasks were both modified from a SRTT task, which was previously involved in tACS experiments (Pollok et al., 2015; Krause et al., 2016). Participants were instructed to press one of four buttons (V, B, N, M) on the keyboard as fast as possible, according to the position of the light rectangles shown on the screen (Figure 1B). The stimulus remained on the screen until the correct response was made. After 500 ms, a new stimulus was displayed. Eight blocks were included, with 120 trials in each block. The locations corresponding to the light rectangles were pseudorandomly distributed in all 8 blocks (R) in RRTT (Figure 1C). The only difference between SRTT and RRTT was that the reactions were not randomized in some SRTT blocks (Figure 1D). The first block and the sixth block were R blocks. In the remaining blocks, the locations of the light rectangles were repeated in a 12-item sequential manner ten times in each block (S). Same to the previous studies (Curran, 1997; Schendan et al., 2003), there were three 12-item second-order predictive sequences (bnmvnbmnvbvm, nvnmbvmnbmvb, mvbmnbnvmbvn) with comparable difficulty assigned to each experimental session in a counterbalanced way across participants. All of the information about the order of the locations was unknown to the participant, which allowed them to acquire the sequence in an implicit manner. The task presentation and the recording of the reaction times (RT) were conducted using E-Prime 2.0 (Psychology Software Tools, Sharpsburg, MD, United States).

### Temporal Interference Stimulation

The TI stimulation was applied by a customized battery-driven stimulator. The instrument performance (the current characterization and the characterization of channel isolation) of this TI stimulator was comparable with that of Grossman et al. (2017) (Supplementary Figure 1 and Supplementary Material 2.1). For safety concerns, currents of all the four output ports were monitored by protecting circuits to ensure security. Once the amplitude of current at one single output port exceeded the safety threshold, the four output ports would all be cut down by relays. The device is powered by batteries and isolated from mains electricity.

**FIGURE 2:**
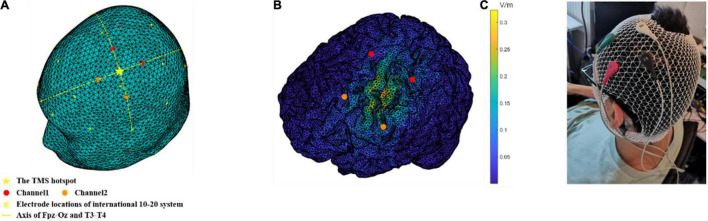
Montage of TI stimulation and the generated electric field on a human head model. **(A)** The stimulation electrodes were located 30 mm away from the TMS hotspot, along the axis of the Fpz-Oz and T3-T4 in the EEG international 10–20 system. **(B)** The distribution of the envelope amplitude along the chosen direction (pointing posterolaterally at a 45° from the mid-line) in the brain. **(C)** An actual scene of electrode placement. The electrodes in red and green constitute channel1 and the electrodes in black and white constitute channel2. The ground electrode (brown) located on the mastoid behind the participant’s left ear to avoid current accumulations due to safety considerations.

The TI stimulation protocol was designed based on simulation analysis using finite element method (FEM) (Supplementary Figure 2 and Supplementary Material 2.2). We used five Ag-AgCl electrodes with a radius of 1 cm (Pistim electrode, Neuroelectrics, Barcelona, Spain), four of which were stimulating electrodes and one was the ground electrode located on the mastoid behind the participant’s left ear to avoid current accumulations due to safety considerations. The stimulation electrodes were located 30 mm away from the FDI hotspot along the axis of the Fpz-Oz and T3-T4 in the EEG 10–20 system (Figure 2). Electrodes were fixed by a medical elastic bandage (Hebei Shengyu Medical Equipment, Hengshui, China) and filled with conductive gel (Quick-Gel, Compumedics USA Inc., Charlotte, CA, United States) to make the impedance of each electrode below 10 kΩ. The stimulation intensity was peak-to-peak 2 mA in a single channel (totally peak-to-peak 4 mA for two channels). Stimulation (20 Hz or 70 Hz) lasted continuously for 30 min. After a 10-min rest since the beginning of stimulation, participant started to perform the motor task, which lasted for 15–20 min (depending on the reaction speed of participants). Participants were asked to rest again until the end of the stimulation. There was a 30 s linearly ramp up and ramp down period at the beginning and the end of the stimulation. For the sham condition, TI stimulation (20 Hz or 70 Hz) only lasted for approximately 60 s (30 s ramp up and 30 s ramp down) at the beginning of this procedure.

### Safety Aspects

After the TI stimulation, we asked the participants to complete a subjective questionnaire (Brunoni et al., 2011; Fertonani et al., 2015), which asked them to rate their sensations including itching, headache, burning, warmth/heat, tingling, metallic/iron taste, fatigue, vertigo, nausea and phosphene during the stimulation and on what extent do they think these feelings were relevant with the stimulation. Participants were asked to rate the extent of these sensations that they experienced, from 0 to 4, representing none, mild, moderate, considerable and strong, respectively. Similarly, the relevance to the stimulation was also rated from 0 to 4, representing none, remote, possible, probable and definite, respectively. Only sensations with a score larger than 1 were taken into consideration.

### Data Analysis

All analyses were performed on MATLAB 2018a (MathWorks, Natick, MA, United States). The mean RT of the correct trials of each block in RRTT or SRTT was calculated. Accuracy was not considered a primary measure because of the ceiling effect (Supplementary Figure 3). Because the calculation of behavior measures needed to integrate the RT of different blocks, any session containing RT of any block beyond 2SD from all participants’ mean RT was removed. The mean RT of all blocks was considered the behavior measure in the RRTT experiment. Motor learning performance (first implicit learning, FIL, Eq. 1; second implicit learning, SIL, Eq. 2) was measured as the RT reduction between S blocks and R blocks in the SRTT experiment.


(1) $$\text{FIL}=RT_{R1}-\left(RT_{S1}+RT_{S2}+RT_{S3}+RT_{S4}\right)/4$$

(2) $$\text{SIL}=RT_{R2}-\left(RT_{S5}+RT_{S6}\right)/2$$

IO curves linearly fitted using the amplitude of MEPs elicited by 100, 110, 120, 130, and 140% RMT were involved in each stimulation condition in the RRTT experiment, and the slope of the IO curve was extracted. Mean MEP amplitudes before and after TI stimulation in each condition were calculated in the SRTT experiment.

Differences in the behavior measures between the stimulation conditions and the control condition (20 Hz vs. sham, 70 Hz vs. sham) were assessed by two-tailed paired *t*-tests. Two 2 (condition: 20 Hz vs. sham/70 Hz vs. sham) × 2 (testing time: before TI stimulation vs. after TI stimulation) repeated measures ANOVA was performed on the slopes of the IO curve and MEP amplitudes. We set age, education level and handedness score as covariables to control their potential influence to the motor cortex excitability (Eisen et al., 1991; De Gennaro et al., 2004; Thomas, 2012). Since there were significant promoting effects found in the behavior measures, slopes of the IO curve and MEP amplitude before and after TI stimulation were compared by one-tailed paired *t*-tests with the hypothesis that MEPs would also be facilitated by TI stimulation. Correlations between the behavior measures and increases in the IO slopes or MEP amplitudes in each condition were tested by two-tailed partial correlations, with age, education level and handedness score controlled as covariables. Bonferroni correction was used to correct for multiple comparisons.

## Results

### Temporal Interference Stimulation at 70 Hz Promoted the Reaction Time and M1 Excitability

**FIGURE 3:**
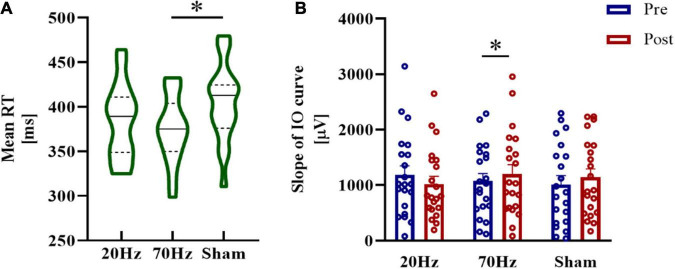
Results of the RRTT experiment. **(A)** The mean RT of the 70 Hz condition was significantly smaller than that of the sham condition. **(B)** Significant increases in the IO slope after TI stimulation were found only in the 70 Hz condition. Error bars represent SEM; *significant at p_corrected_ < 0.05.

In the RRTT experiment, the stimulation condition of 70 Hz showed the lowest mean RT, which was significantly different from the sham condition (*t* = −2.953, p_corrected_ = 0.019, Cohen’s *d* = 0.716) (Figure 3A). There was no significant difference in the comparison between the 20 Hz and sham groups (*t* = −1.199, p_corrected_ = 0.498).

For the slope of the IO curve, we found no significant results either in the main effects of condition or the main effects of testing time or the interaction of the comparison between 70 and 20 Hz with sham (all *p*s > 0.05). Paired *t*-tests revealed a significant increase in the IO slope after TI stimulation at 70 Hz (70 Hz: *t* = 2.395, p_corrected_ = 0.040, Cohen’s *d* = 0.523, one-tailed) but not at 20 Hz or in the sham condition (20 Hz: *t* = −1.075, p_corrected_ = 0.443, one-tailed; sham: *t* = 1.597, p_*corrected*_ = 0.189, one-tailed) (Figure 3B).

### Temporal Interference Stimulation at 20 Hz Improved Implicit Motor Learning and MEP Amplitude

**FIGURE 4:**
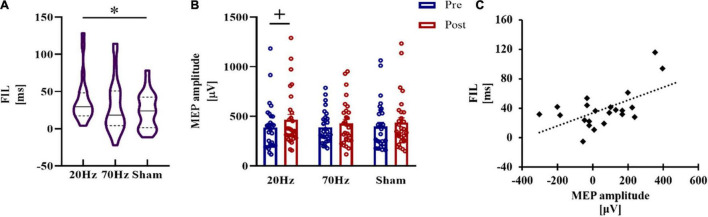
Results of the SRTT experiment. **(A)** Implicit motor learning during FIL in SRTT. A significant RT reduction was only obtained in the 20 Hz condition. **(B)** Marginally significant increases in MEP amplitude after TI stimulation at 20 Hz. **(C)** RT reduction of FIL and the MEP increase in the 20 Hz condition was significantly positively correlated in the 20 Hz condition. Error bars represent SEM; + marginally significant at 0.05 < p_corrected_ < 0.1, *significant at p_corrected_ < 0.05.

In the SRTT experiment, TI stimulation at 20 Hz showed the highest RT reduction in FIL, which was significantly different from the sham condition, while another comparison did not show significance (20 Hz vs. sham: *t* = 2.577, p_corrected_ = 0.041, Cohen’s *d* = 0.625; 70 Hz vs. sham: *t* = 0.197, p_corrected_ = 1) (Figure 4A). No significant differences were found in SIL between the stimulation conditions and sham conditions (20 Hz vs. sham: *t* = 0.5116, p_corrected_ = 1; 70 Hz vs. sham: *t* = 1.5716, p_corrected_ = 0.269).

For MEP amplitude, when comparing the 20 Hz condition and sham condition, repeated measures analysis of variance (ANOVA) revealed a significant main effect of testing time (*F* = 4.230, *p* = 0.050, η^2^ = 0.145), while the main effect of condition (*F* = 1.463, *p* = 0.238) and the interaction (*F* = 0.345, *p* = 0.563) were not significant. There was also a significant main effect of testing time (*F* = 6.523, *p* = 0.017, η^2^ = 0.207) in the comparison between 70 Hz and sham, and no significant result was found in the main effect of condition (*F* = 2.028, *p* = 0.167) or in the interaction (*F* = 0.942, *p* = 0.341). MEP amplitudes increased after 20 Hz TI stimulation compared with MEP measured before stimulation at a marginally significant level (*t* = 2.137, p_corrected_ = 0.062, Cohen’s *d* = 0.397, one-tailed) (Figure 4B). The increase in MEP amplitudes in the 70 Hz and sham conditions was not significant (70 Hz: *t* = 1.570, p_corrected_ = 0.192; sham: *t* = 1.254, p_corrected_ = 0.330).

The significant reduction in RT during FIL in the 20 Hz condition was positively correlated with the MEP increase (*r* = 0.580, p_corrected_ = 0.027) (Figure 4C), while RT reductions in the other two conditions showed no significant correlations with the MEP increase (70 Hz: *r* = 0.073, p_corrected_ = 1; sham: *r* = −0.360, p_corrected_ = 0.426).

### Temporal Interference Stimulation Caused Minor Side Effects on Participants

Side effects occurring during TI stimulation were minor and tolerable according to the participants’ descriptions and our observations. In the RRTT experiment, there were 2 sessions that participants reported moderate and larger discomforts (2.38% in active sessions (20 and 70 Hz), 4.76% in sham sessions, χ^2^ = 0.258, *p* = 0.611). While in the SRTT experiment, there were 4 sessions that participants reported moderate and larger discomforts (5.17% in active sessions, 3.45% in sham sessions, χ^2^ = 0.131, *p* = 0.717). More details are given in Tables 1, 2. Notably, all discomforts during the sham sessions occurred in the middle of the session or at the end of the session, which could imply that sham stimulation did not directly cause the sensations. Our subsequent investigations of the participants also reported no other side effects after the experiments.

**TABLE 1:** Discomforts in RRTT.

Sensations	Active sessions (N/42)	Sham sessions (N/21)
None	41	20
Fatigue and Vertigo	0	1
Headache	1	0

**TABLE 2:** Discomforts in SRTT.

Sensations	Active sessions (N/58)	Sham sessions (N/29)
None	55	28
Fatigue	3	1

## Discussion

In this study, we applied TI stimulation to healthy human participants to explore the modulatory effects of TI stimulation. We investigated the changes in motor performance resulting from TI stimulation applied over M1 in two experiments involving different motor tasks. TI stimulation with an envelope frequency of 70 Hz promoted the RT performance of the motor task compared with the sham condition in the RRTT experiment. TI stimulation with an envelope frequency of 20 Hz applied over M1 enhanced the FIL performance compared with sham stimulation, and the performance was positively correlated with the MEP increase in the SRTT experiment.

### Temporal Interference Stimulation Is Effective in the Human Motor Cortex

Our study, for the first time, suggests that the idea of TI stimulation is plausible, not only in computational models and experiments on mice (Grossman et al., 2017; Fariba et al., 2019; Huang and Parra, 2019; Rampersad et al., 2019; Huang et al., 2020; Lee et al., 2020), but also in actual experiments performed on healthy human participants. Since the idea of TI stimulation has been raised, the only *in vivo* investigations have been performed on mouse brains (Grossman et al., 2017). The human brain is much larger, and the layers around the brain in humans are thicker, which causes up to 100 times weaker electric fields in the human brain than in the mouse brain at the same stimulation intensity (Alekseichuk et al., 2019). The stimulation waveform of TI stimulation is an envelope-modulated waveform produced by the superposition of two sine waves, which has not been previously tested on humans. Envelope-tACS using only envelope waveforms of speech without carrier waves have been used to improve speech perception and processing (Riecke et al., 2018; Wilsch et al., 2018; Kadir et al., 2020), but the effects are still controversial (Asamoah et al., 2019; Erkens et al., 2020; Kosem et al., 2020). Amplitude-modulated tACS (AM-tACS) was proposed as a promising way to allow for effective magnetoencephalography (MEG) or EEG signal reconstruction during electrical stimulation (Witkowski et al., 2016; Kasten et al., 2018; Negahbani et al., 2018; Haslacher et al., 2021). However, similar to TI stimulation, studies on AM-tACS have also mostly focused on simulations, and no systematic experimental test to validate the effectiveness of AM-tACS on humans has been performed. Whether envelope modulated waveforms have comparable effects to conventional tACS is unknown.

To solve these problems, we applied TI stimulation to the human M1 area and found some significant effects. In the RRTT experiment, only 70 Hz TI stimulation promoted RT performance and motor cortical excitability. In the SRTT experiment, only 20 Hz TI stimulation increased the first implicit motor learning and MEP amplitudes. We found significant main effects of testing time in the analysis of MEP amplitude, which might indicate a training effect of the motor learning task (Classen et al., 1998; Fattinger et al., 2017). But only the correlation between FIL and MEP increase in 20 Hz condition was significant, indicating the increase of motor cortex excitability related with TI stimulation only occurred in 20 Hz TI stimulation. This can be supported by a meta-analysis, which shows that exogenously applied electric fields in beta frequency range can increase motor cortex excitability (Wischnewski et al., 2019). The time delay between the end of TI stimulation and the following MEP measurement was about 5–10 min, within the time window of the aftereffect of conventional tES (Kuo et al., 2013; Kasten et al., 2016). Future studies could explore the duration of the aftereffect with parameterized time intervals between the end of the TI stimulation and the following measurements.

However, the effect of TI stimulation shown in this study is not as phenomenal as that in the mice study. Future studies could explore the mechanisms of TI stimulation at the level of brain regions and networks by corresponding neuroimaging techniques, e.g., functional magnetic resonance imaging (fMRI), and try to build a more effective TI stimulation system for human with a better understanding of it.

### High Gamma and Beta Oscillations May Represent Different Motor Functions in M1

Distinct effects of 20 and 70 Hz TI stimulation may indicate different functions of these two motor cortical oscillations. High gamma and beta are considered vital neural rhythms corresponding to the activation of M1 (Cheyne et al., 2008; Muthukumaraswamy, 2010; Gaetz et al., 2013; Espenhahn et al., 2019, 2020; Haar and Faisal, 2020). TACS (70 Hz) has been reported to increase motor velocity and motor acceleration during stimulation in visually guided motor tasks (Joundi et al., 2012; Moisa et al., 2016). Meanwhile, tACS at 20 Hz has been reported to improve the performance of implicit learning of SRTT in previous studies (Pollok et al., 2015; Krause et al., 2016). These findings could imply that beta and high-gamma neural rhythms predominate in different motor functions in M1. Our results duplicate the functional separation between brain oscillations at 20 and 70 Hz. The functional separation between 70 Hz (2,000 and 2,070 Hz) and 20 Hz (2,000 and 2,020 Hz) TI stimulation also supports the hypothesis that electric fields of high-frequency carriers (2,000 Hz) may have little contribution to the results because of the intrinsic feature of the neural membrane that filters electrical signals in a low-pass manner (Hutcheon and Yarom, 2000; Esmaeilpour et al., 2020). Additional studies could explore the effects of carrier frequency and envelope frequency more deeply. For instance, future studies could stimulation deep brain regions and monitor if there is some influence of the stimulation on the superficial brain regions. So that whether the single high-frequency stimulation could influence the neural activity of the human brain can be explored.

### The Application Potential of Temporal Interference Stimulation as a Non-invasive Brain Stimulation Technique

We assessed the side effects of TI stimulation by subjective reporting of the participants. In most sessions (>95%), participants reported no obvious side effects. No sensations related to the skin, such as tingling, itching and burning, were reported, and no burns or reddening of the skin were observed by the experimenters. Only fatigue, vertigo and headache were reported in several sessions, including two sham sessions. The side effects reported by participants in this study were far less than those reported for conventional tES (Brunoni et al., 2011; Fertonani et al., 2015; Matsumoto and Ugawa, 2017), which indicates that TI stimulation may have advantages over conventional tES in safety, user-friendliness and blinding performance.

Our study indicates that TI stimulation can be used as a new technique to modulate human neural activities in a non-invasive way. The TI stimulation was aimed to stimulate deep brain regions. However, we only explored the effects of TI stimulation on human M1 in the present study. Previous studies have suggested that to achieve effect comparable with DBS, the stimulation protocol might need to be modified to improve the stimulation strength and focality (Rampersad et al., 2019). Stimulation effects on other deeper brain regions with more sophisticated functions rely on a better understanding of the working mechanisms and prospects of TI stimulation in humans, which needs to be explored in additional research utilizing combinations of neuron models, finite element modeling simulations and experiments (Rampersad et al., 2019). There are a lot of technical problems unresolved. Therefore, before exploring the effectiveness of TI stimulation in deep brain regions, we should firstly test whether this new stimulation technique could influence human brain activity of the superficial cortex. Otherwise if TI stimulation doesn’t show effects in deep brain regions, we could not explain the reason, e.g., the physical property of stimulation or other technical issues. Anyway, future studies should explore the effect of TI stimulation in deep brain regions and promote the applications of TI stimulation in clinical practice.

## Conclusion

Our study reveals the promoting effect of TI stimulation on human motor functions and motor cortex excitability. TI stimulation with different envelope frequencies showed separate promoting effects on different motor tasks, which implied that TI stimulation may work through a low-frequency envelope. Future investigations of TI stimulation in humans could explore stimulation effects in deeper brain regions under the guidance of modeling works. In summary, TI stimulation could be a promising new technique for non-invasive brain stimulation in humans with clinical application potentials.

## Data Availability Statement

The datasets presented in this study can be found in online repositories. The names of the repository/repositories and accession number(s) can be found below: cnp.ustc.edu.cn.

## Ethics Statement

The studies involving human participants were reviewed and approved by the Human Ethics Committee of the University of Science and Technology of China. The patients/participants provided their written informed consent to participate in this study.

## Author Contributions

RM: project administration, methodology, software, formal analysis, investigation, data curation, writing – original draft, and visualization. XX: methodology, hardware testing, formal analysis, investigation, data curation, writing – original draft, and visualization. WZ and HS: methodology, software, writing, review, and editing. ZL: methodology, hardware design and implementation, and hardware testing. QW: formal analysis, investigation, and data curation. JC: methodology, validation, and hardware testing. CF, XC, and RZ: writing, review, and editing. JW and G-JJ: methodology and techniques of TMS. XW and BQ: methodology, hardware, and supervision. XZ: conceptualization, funding acquisition, methodology, writing, review, editing, and supervision. All authors contributed to the article and approved the submitted version.

## Conflict of Interest

The authors declare that the research was conducted in the absence of any commercial or financial relationships that could be construed as a potential conflict of interest.

## Publisher’s Note

All claims expressed in this article are solely those of the authors and do not necessarily represent those of their affiliated organizations, or those of the publisher, the editors and the reviewers. Any product that may be evaluated in this article, or claim that may be made by its manufacturer, is not guaranteed or endorsed by the publisher.
